# Supplementary material for: The interactome of intact mitochondria by cross-linking mass spectrometry provides evidence for coexisting respiratory supercomplexes
Source: Mol Cell Proteomics. 2017 Dec 8;17(2):216–32. doi: 10.1074/mcp.RA117.000470 (PMC5795388; doi:10.1074/mcp.RA117.000470)
Supplement: Supplemental Data [file supp_RA117.000470_133922_0_supp_23978_fzffwf.zip › spectra_annotation/mito_spectra_annotation/95-1-12-1-5-1.pdf]

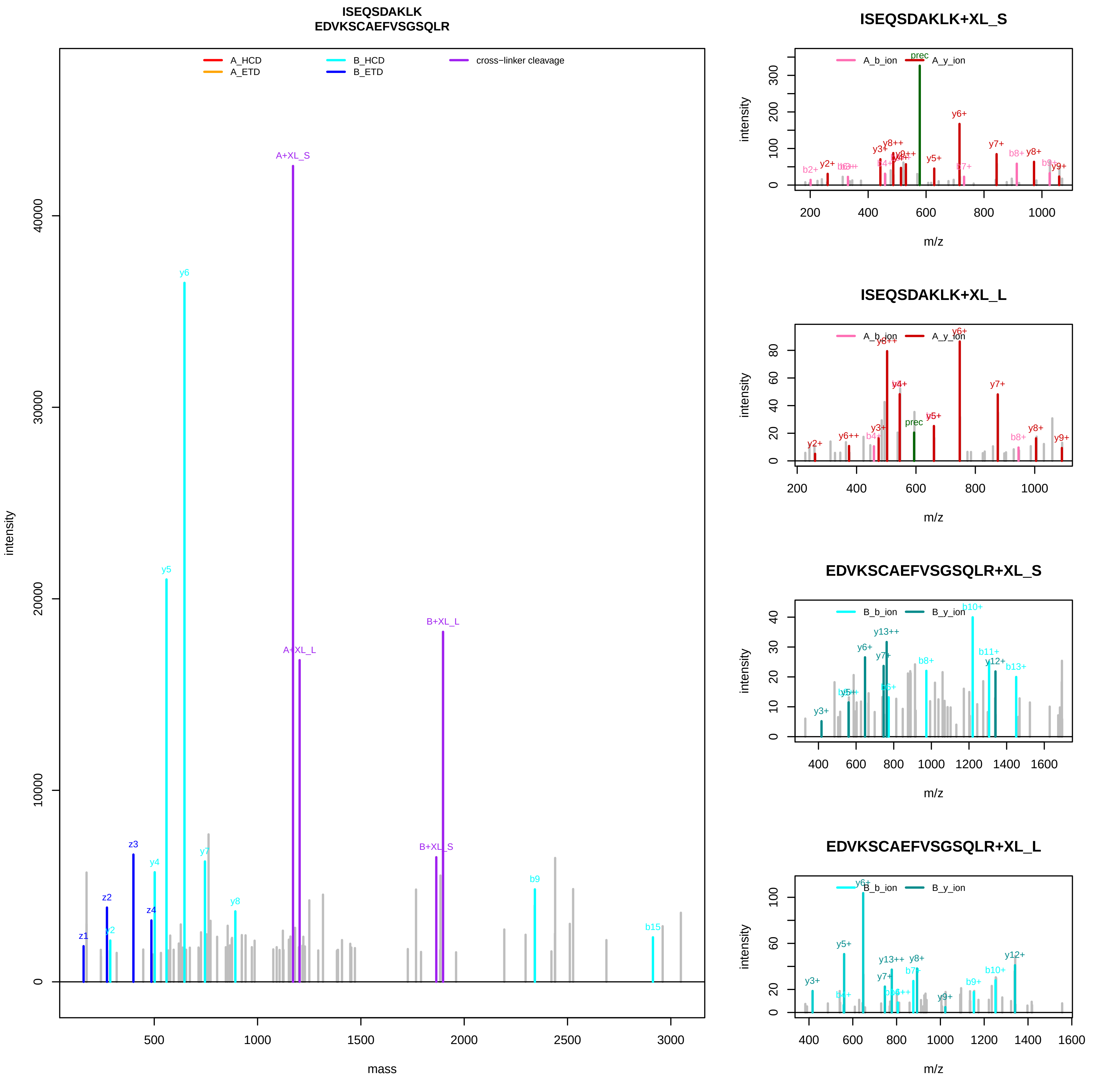

ISEQSDAKLK
EDVKSCAEFVSGSQLR
A_HCD
A_ETD
B_HCD
B_ETD
cross−linker cleavage
A+XL_S
A+XL_L
B+XL_L
B+XL_S
y6
y5
y4
y7
y8
y2
z1
z2
z3
z4
b9
b15
intensity
mass
ISEQSDAKLK+XL_S
A_b_ion
A_y_ion
prec
y6+
y7+
y5+
y8+
b8+
b2+
y2+
m/z
ISEQSDAKLK+XL_L
EDVKSCAEFVSGSQLR+XL_S
B_b_ion
B_y_ion
b10+
y13++
b8+
b11+
y12+
b13+
y3+
EDVKSCAEFVSGSQLR+XL_L
y6+
y5+
y12+
b10+
b9+
